# Effect of dominant cement distribution zone on pain relief after unipedicular percutaneous vertebroplasty

**DOI:** 10.3389/fsurg.2026.1806822

**Published:** 2026-04-08

**Authors:** Murat Özcan Yay, Melih Çetiner

**Affiliations:** 1 Department of Neurosurgery, Aydın Adnan Menderes University Faculty of Medicine, Aydın, Türkiye; 2 Department of Neurosurgery, Aydın State Hospital, Aydın, Türkiye

**Keywords:** cement, osteoporotic fractures, pain measurement, spinal fractures, vertebroplasty

## Abstract

**Background:**

Cement location within the vertebral body may influence pain relief after unipedicular vertebroplasty. We tested a simple four-zone classification of dominant intravertebral cement distribution in osteoporotic vertebral compression fractures.

**Methods:**

We retrospectively analyzed 425 patients treated from 2021 to 2024. On postoperative imaging, the vertebral body was divided into four equal zones (Zones 1–4) and the zone with the greatest cement accumulation was recorded. Pain was measured with the visual analog scale (VAS) before and after the procedure; change in VAS was the primary endpoint. Multivariable linear regression modeled change in VAS. Logistic regression modeled clinical response (change in VAS > 4).

**Results:**

Mean VAS decreased from 7.63 ± 0.84 to 3.31 ± 1.06 (*p* < 0.001), with mean change in VAS of 4.32 ± 1.38. Change in VAS differed across zones (*p* < 0.001), highest in Zone 4 and lowest in Zone 1. Complications occurred in 45.4% (primarily cement leakage) without permanent neurological deficit. In linear regression, dominant zone independently predicted change in VAS (B = 0.852; standardized *β* = 0.546; *p* < 0.001) and overall fit was strong (R² = 0.724; adjusted R² = 0.717). In logistic regression (*n* = 387), Omnibus *χ*² = 280.646 (df = 13, *p* < 0.001) and Nagelkerke R² = 0.729; zone, preoperative VAS, and cement volume were independent predictors. Calibration was acceptable (Hosmer–Lemeshow *p* = 0.941). Compared with Zone 4, Zones 1–3 showed lower odds of response; higher baseline VAS increased the odds.

**Conclusions:**

Dominant cement zone strongly predicts pain improvement after unipedicular vertebroplasty and may serve as a practical procedural quality marker.

## Introductıon

Osteoporotic vertebral compression fractures are a common consequence of osteoporosis and an important driver of pain, reduced mobility, loss of independence, and impaired health related quality of life in older adults. Beyond acute symptoms, vertebral fractures are also linked to longer term complications such as progressive sagittal imbalance and hyperkyphosis, and they have been associated with increased morbidity and mortality (1–3).

Percutaneous vertebroplasty has been widely used to provide rapid pain relief and mechanical stabilization by injecting polymethylmethacrylate cement into the fractured vertebral body. At the same time, its clinical effectiveness has remained debated because randomized sham controlled trials and evidence syntheses have shown mixed results, particularly when vertebroplasty is compared with placebo type procedures rather than with usual care. Despite this controversy, contemporary reviews continue to emphasize that vertebroplasty may still have a role in carefully selected patients, which has increased interest in identifying technical and patient related factors that could explain heterogeneity in outcomes and help optimize results (4–7). Recent high-quality evidence syntheses comparing minimally invasive augmentation techniques also indicate that clinical benefit and complication profiles may differ across approaches, supporting ongoing efforts to refine patient selection and procedural targets (8).

One procedural variable that has gained attention is the choice of access route. Unipedicular vertebroplasty and kyphoplasty approaches have been increasingly adopted because they may shorten procedure time and reduce radiation exposure while achieving pain and functional outcomes comparable to bipedicular techniques in many series and meta analyses. Nevertheless, unipedicular techniques can produce more variable cement spread within the vertebral body, and there is no universal agreement on what constitutes an optimal intravertebral cement location when only one pedicle is used (9, 10). Accordingly, simple and reproducible descriptors of intravertebral cement location are needed to standardize reporting and to guide technical optimization in unipedicular procedures (11).

Beyond the absolute volume of injected cement, the spatial distribution pattern of cement has been repeatedly proposed as a determinant of biomechanical stability and clinical response. Clinical studies have suggested that more diffuse, trabecular filling patterns can be associated with better pain relief and functional recovery than compact, block like filling patterns. A recent systematic review of cement distribution classifications also concluded that sufficient and more even cement distribution is related to better radiographic restoration and pain outcomes, and that cement contact with both endplates may reduce refracture risk (12, 13). However, existing distribution classifications are heterogeneous and not uniformly applicable in daily practice, which limits comparability across studies and reduces bedside usability (13). Complementary biomechanical work supports the concept that where cement is positioned within the vertebral body can meaningfully alter load transfer and mechanical behavior, reinforcing the idea that distribution is not simply an imaging descriptor but a potentially actionable technical target (14, 15).

Cement distribution is also clinically relevant because it intersects with safety. Cement extravasation is common and is usually well tolerated, yet it remains the major source of clinically important complications, including neural compression when leakage tracks toward the foramina or canal, and cement pulmonary embolism when leakage occurs via venous pathways. More recent reviews have suggested that although vertebroplasty may produce more leaks than some alternative augmentation techniques, many leaks are clinically silent, which further emphasizes the need to distinguish leakage incidence from leakage that influences symptoms or outcomes (16, 17). Therefore, distribution targets should ideally optimize analgesic benefit without increasing clinically relevant leakage (18).

In parallel with technical optimization, there is growing interest in patient related biological factors that could modulate pain trajectories and recovery. Systemic inflammatory markers derived from routine blood counts, including the neutrophil to lymphocyte ratio, have been studied in osteoporotic vertebral fracture populations and in patients undergoing cement augmentation procedures, although available results are heterogeneous and the prognostic value of these markers remains uncertain. Importantly, the interaction between intravertebral cement distribution patterns and laboratory markers has been insufficiently examined, leaving open the question of whether distribution related outcome differences are purely mechanical or partly reflect underlying biological context (19). This gap further supports examining cement distribution together with routinely available laboratory markers within a single adjusted framework.

Against this background, we designed the present study to evaluate, in a large cohort of patients treated with unipedicular percutaneous vertebroplasty for osteoporotic vertebral compression fractures, whether the dominant intravertebral cement location assessed using a standardized four zone classification predicts pain outcomes. We specifically aimed to quantify differences in pain improvement across Zones 1 to 4 and to determine whether zonal distribution independently predicts clinically meaningful pain reduction after adjustment for cement volume and relevant clinical, radiological, and laboratory variables.

## Materials and methods

### Study design and ethics

This single-center, retrospective, non-interventional observational study evaluated patients with osteoporotic vertebral compression fractures who underwent unipedicular percutaneous vertebroplasty. The study was conducted after approval from the Aydın Adnan Menderes University Non-Interventional Clinical Research Ethics Committee (Decision:03, Protocol no:2026/31) and in accordance with the Declaration of Helsinki. All data were obtained from existing hospital records and were analyzed in an anonymized manner.

### Study setting and population

Hospital records were retrospectively reviewed for patients treated at Aydın State Hospital between 2021 and 2024. During this period, 425 patients who underwent unipedicular percutaneous vertebroplasty for osteoporotic vertebral compression fractures were identified and included in the study cohort.

### Eligibility criteria

Patients were eligible if they had a diagnosis of osteoporotic vertebral compression fracture, underwent unipedicular percutaneous vertebroplasty, and had available preoperative and postoperative visual analog scale (VAS) pain scores. Availability of preoperative neutrophil-to-lymphocyte ratio (NLR) and serum calcium measurements was also required. Patients with vertebral fractures related to tumor or infection were excluded. Because of the retrospective nature of the study, fracture acuity and MRI bone marrow edema status were not uniformly available in a sufficiently standardized manner and therefore were not used as formal eligibility criteria. Patients with missing key clinical, radiological, or laboratory data in the hospital records were excluded from analyses requiring those variables.

### Clinical and demographic variables and outcome measures

Age, sex, and comorbidities were extracted from medical records. Pain intensity was assessed using the VAS before the procedure and after the procedure as documented in routine postoperative clinical evaluation. Postoperative VAS was extracted from the first routine clinical assessment after the procedure as documented in the medical records. When more than one postoperative pain score was available, the earliest recorded assessment was used. The present analysis focused on early postoperative pain outcomes, and standardized long-term follow-up measurements were not consistently available for all patients. Pain improvement was quantified as change in VAS, calculated as the difference between preoperative and postoperative VAS scores (preoperative minus postoperative). Preoperative VAS, postoperative VAS, and change in VAS were used for group comparisons and regression analyses as specified below.

### Fracture characteristics

Fracture level and fracture type were determined using preoperative imaging available in the records. Fractures were categorized according to the AO Spine fracture classification as A1, A2, A3, or A4. Fracture level was recorded as thoracic or lumbar.

### Percutaneous vertebroplasty technique

All procedures were performed using a unipedicular transpedicular approach under fluoroscopic guidance and local anesthesia with a standardized technique. After advancement of the working cannula through the pedicle into the fractured vertebral body, polymethylmethacrylate (PMMA) cement was injected in a controlled manner after reaching an appropriate viscosity. Cement injection was performed under continuous fluoroscopic monitoring to achieve satisfactory intravertebral filling while minimizing the risk of posterior wall extravasation or venous leakage, and the injection was terminated when adequate filling was achieved or leakage was suspected on real-time imaging. Postoperative imaging evaluation was performed immediately after the procedure according to institutional routine, and these images were used to assess cement distribution and procedure-related complications. The total injected cement volume (cc) was recorded for each patient.

### Assessment of zonal cement distribution

**Figure 1:**
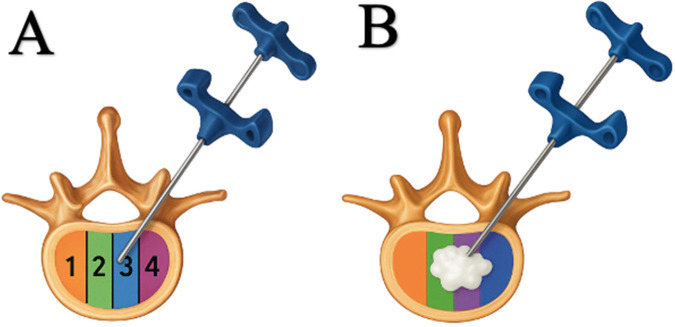
Four-zone classification for intravertebral cement distribution in unipedicular vertebroplasty.

Intravertebral cement distribution was assessed on postoperative imaging. As illustrated in Figure 1A, the vertebral body was divided into four equal mediolateral zones (Zones 1–4). Zones were numbered consistently across all cases using a standardized orientation on the postoperative image. The dominant cement zone was defined as the zone containing the greatest cement accumulation on the image demonstrating maximal cement spread (Figure 1B). In cases where cement extended into more than one zone, the zone containing the largest proportion of cement was considered dominant.

For cement distribution assessment, the vertebral body was evaluated on the anteroposterior postoperative imaging view, and its mediolateral width was divided into four equal zones from one lateral cortical margin to the other. Zoning was performed using the full mediolateral vertebral body width as the reference axis, defined by the lateral cortical margins, without relying on pedicular landmarks or vertebral midline estimation. Accordingly, Zones 1 and 4 represented the outer lateral portions, whereas Zones 2 and 3 represented the more central mediolateral portions of the vertebral body.

The dominant cement zone was defined as the zone containing the visually greatest proportion of cement distribution within the vertebral body. No fixed quantitative threshold (e.g., ≥50%) was applied; instead, dominance was determined by visual predominance to ensure practical applicability in routine clinical settings. This classification was intended as a simple and clinically applicable method for standardized assessment of mediolateral cement location in unipedicular vertebroplasty.

**Figure 2:**
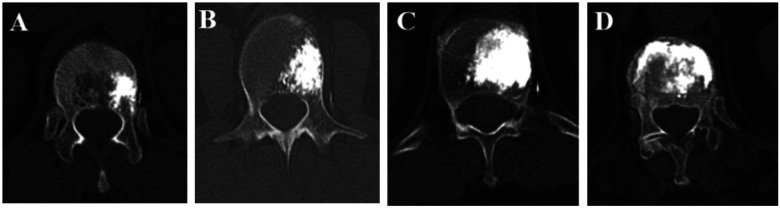
Epresentative axial imaging examples of dominant cement distribution zones (zones 1–4) following unipedicular percutaneous vertebroplasty. **(A–D)** Illustrate typical patterns of cement predominance used for classification, facilitating practical application of the four-zone system in routine clinical settings.

The same proportional zoning method was applied uniformly across thoracic and lumbar vertebrae without segment-specific modification. The dominant cement zone was assessed independently by two observers based on the postoperative imaging set. Interobserver agreement for the four-zone classification was evaluated using Cohen's kappa coefficient. Representative examples of each dominant cement distribution zone are provided in Figure 2.

### Laboratory parameters

Preoperative neutrophil and lymphocyte counts obtained from routine blood tests were used to calculate the neutrophil-to-lymphocyte ratio (NLR). Serum calcium levels measured preoperatively were also recorded.

### Complications

**Figure 3:**
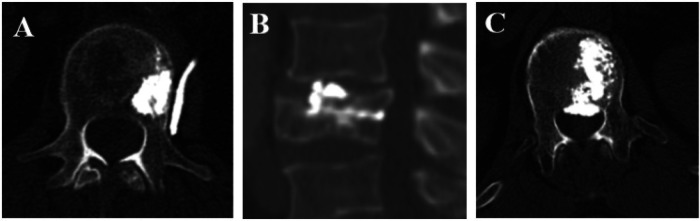
Representative examples of cement leakage patterns observed after unipedicular vertebroplasty. **(A)** Asymptomatic venous leakage, **(B)** intradiscal leakage, and **(C)** asymptomatic epidural leakage.

Procedure-related complications were assessed retrospectively using postoperative imaging records together with routine clinical documentation. Thus, complications were not defined solely by symptomatic events but also included radiographically detected events documented in the available postoperative imaging records. Cement leakage, when identifiable on postoperative imaging, was recorded as present or absent and, when available, further categorized according to its distribution pattern (for example, intradiscal, venous, spinal canal, or combined). Because this was a retrospective study based on routine institutional practice, complication detection reflected the available postoperative imaging records rather than a study-mandated uniform CT protocol. Representative examples of different cement leakage patterns are shown in Figure 3.

### Statistical analysis

Statistical analyses were performed using IBM SPSS Statistics (IBM Corp., Armonk, NY, USA). Continuous variables are presented as mean ± standard deviation or median (interquartile range), as appropriate, and categorical variables as counts and percentages. Differences in change in VAS among the four cement distribution zones were assessed using one-way analysis of variance (ANOVA) for normally distributed variables; when the overall ANOVA was significant, pairwise comparisons were performed using Tukey's honestly significant difference test. For non-normally distributed variables, the Kruskal–Wallis test was used, followed by Mann–Whitney *U*-tests for *post hoc* pairwise comparisons when indicated. To evaluate whether dominant zonal cement distribution independently predicted pain improvement, a multivariable linear regression model was constructed adjusting for age, sex, fracture type, fracture level, preoperative VAS score, and cement volume. Because AO type A4 fractures were rare, AO fracture morphology was additionally analyzed using pooled severity categories to improve model stability: A1–A2 were grouped as less severe and A3–A4 as more severe fractures. This pooled AO severity variable was included in both linear and logistic regression models. As a secondary analysis, a multivariable logistic regression model was developed to assess clinical response, defined as change in VAS ≥ 4. The logistic regression analysis was conducted using complete-case analysis; therefore, patients with missing values in one or more model variables were excluded from that analysis. A two-sided *p* value < 0.05 was considered statistically significant. Bonferroni-adjusted significance threshold was set as 0.05/number of comparisons.

## Results

### Patient characteristics

A total of 425 patients who underwent unipedicular percutaneous vertebroplasty for osteoporotic vertebral compression fractures were included. The demographic and clinical characteristics of the cohort are summarized in Table 1. The mean age was 68.56 ± 11.15 years; 307 patients (72.2%) were female and 118 (27.8%) were male. Fractures were located in the lumbar region in 275 patients (64.7%) and in the thoracic region in 150 patients (35.3%). According to the AO classification, fracture types were distributed across A1, A2, A3, and A4 categories (Table 1).

**Table 1:** Baseline demographic, clinical, and procedural characteristics of the study cohort.

Variable	Total (*n* = 425)
Age (years), mean ± SD	68.56 ± 11.15
Sex, *n* (%)	
Female	307 (72.2%)
Male	118 (27.8%)
Fracture level, *n* (%)	
Thoracic	150 (35.3%)
Lumbar	275 (64.7%)
AO fracture type, *n* (%)	
A1	170 (40.0%)
A2	25 (5.88%)
A3	226 (53.18%)
A4	4 (0.94%)
Preoperative VAS, mean ± SD	7.63 ± 0.84
Postoperative VAS, mean ± SD	3.31 ± 1.06
Change in VAS, mean ± SD	4.32 ± 1.38
Cement volume (cc), mean ± SD	3.11 ± 0.71
NLR, median (IQR_25_-IQR_75_)	2.71 (1.76–5.00)
Serum calcium (mg/dL), median (IQR_25_-IQR_75_)	9.1 (8.3–9.5)
Complications, *n* (%)	193 (45.4%)

AO, Arbeitsgemeinschaft für Osteosynthesefragen; VAS, visual analog scale; NLR, neutrophil-to-lymphocyte ratio.

### Clinical outcomes

A significant reduction in pain scores was observed after percutaneous vertebroplasty in the overall cohort. The mean preoperative VAS score was 7.63 ± 0.84, decreasing to 3.31 ± 1.06 postoperatively. The change in pain score (change in VAS) was statistically significant (*p* < 0.001).

### Clinical outcomes according to zonal cement distribution

Patients were categorized into four groups based on the dominant intravertebral cement zone (Zones 1–4). Interobserver agreement for the dominant cement zone classification was high (Cohen's kappa = 0.84), supporting the reproducibility of the four-zone system. Zone-based clinical outcomes are presented in Table 2. Significant between-group differences were observed for preoperative VAS, postoperative VAS, and change in VAS (all *p* < 0.001).

**Table 2:** Comparison of clinical outcomes, cement volume, laboratory parameters, and complications across cement distribution zones.

Parameter	Zone 1 (*n* = 22)	Zone 2 (*n* = 88)	Zone 3 (*n* = 163)	Zone 4 (*n* = 150)	*p*
Preoperative VAS, mean ± SD	7.05 ± 0.72^a^	7.11 ± 0.78^a^	7.64 ± 0.75^b^	8.01 ± 0.78^c^	**<0**.**001**^1^
Postoperative VAS, mean ± SD	4.64 ± 0.90^a^	4.24 ± 0.83^a^	3.36 ± 0.85^b^	2.53 ± 0.69^c^	**<0**.**001**^1^
Change in VAS, mean ± SD	2.41 ± 0.73^a^	2.87 ± 0.67^a^	4.29 ± 0.93^b^	5.48 ± 1.00^c^	**<0**.**001**^1^
Cement volume (cc), mean ± SD	2.55 ± 0.59^a^	2.70 ± 0.70^a^	3.10 ± 0.63^b^	3.44 ± 0.64^c^	**<0**.**001**^1^
Serum calcium (mg/dL), median (IQR_25_-IQR_75_)	8.5 (4.5–9.3)	9.2 (5.3–9.5)	9.1 (8.3–9.5)	9.2 (8.4–9.7)	0.024^2^
NLR, median (IQR_25_-IQR_75_)	3.67 (2.43–8.42)	2.69 (1.90–4.27)	2.74 (1.75–4.59)	2.58 (1.72–5.44)	0.043^2^
Complications, *n* (%)	8 (36.4)	48 (54.5)	75 (46.0)	62 (41.3)	0.195^3^

Values with different superscript letters (a–c) are significantly different in *post-hoc* pairwise comparisons according to the Bonferroni-adjusted threshold. VAS, visual analog scale; NLR, neutrophil-to-lymphocyte ratio.

The bold values indicate statistically significant results, defined as *p* < 0.05.

^1^ One-way ANOVA test and *post-hoc* Tukey test were used.

^2^ Kruskall-Wallis test and Mann–Whitney *U*-test were used.

^3^ Pearson chi-square test was used. For pairwise comparisons among four zones, Bonferroni correction was applied (adjusted significance threshold *p* < 0.0083, i.e., 0.05/6).

Post-hoc analyses showed that change in VAS was significantly higher in Zones 3 and 4 compared with Zones 1 and 2 (*p* < 0.05). The greatest pain reduction was observed in Zone 4, whereas the lowest change in VAS was noted in Zone 1. When zones were regrouped as central (Zones 2 and 3) and peripheral (Zones 1 and 4), change in VAS values were significantly higher in the peripheral zone group compared with the central zone group (*p* < 0.001).

### Relationship between cement volume and change in VAS

Cement volume showed a weak-to-moderate positive correlation with change in VAS (r = 0.404, *p* < 0.001) (Table 3). Cement volume also differed significantly across zonal distribution groups (*p* < 0.001). Zone 4 had a significantly higher cement volume than the other zones, and Zone 3 had a significantly higher cement volume than Zones 1 and 2. No significant difference in cement volume was observed between Zones 1 and 2 (Table 2).

**Table 3:** Multivariable linear regression analysis for predictors of change in VAS.

Variable	B coefficient	ß	95% CI for B	*p*-value
Zone	0.851	0.545	0.751–0.951	**<0.001**
Age	0.000	−0.002	−0.007–0.007	0.946
Sex	0.072	0.023	−0.098–0.241	0.408
AO severity (pooled)	0.226	0.082	0.072–0.380	**0.004**
Fracture level (T/L)	−0.039	−0.014	−0.196–0.118	0.623
Preoperative VAS	0.656	0.401	0.559–0.754	**<0.001**
Cement volume (cc)	0.138	0.072	0.025–0.252	**0.017**
NLR	0.012	0.044	−0.003–0.027	0.128
Serum calcium (mg/dL)	0.005	0.079	0.002–0.009	**0.004**

Linear regression was used. AO fracture morphology was analyzed using pooled severity categories (A1–A2 = less severe; A3–A4 = more severe). VAS, visual analog scale; NLR, neutrophil-to-lymphocyte ratio; T/L, thoracic/lumbar.

The bold values indicate statistically significant results, defined as *p* < 0.05.

### Laboratory parameters

Preoperative NLR and serum calcium levels were evaluated for completeness. Neither NLR nor serum calcium showed a meaningful correlation with change in VAS (NLR: r = −0.061, *p* = 0.218; serum calcium: r = 0.088, *p* = 0.079).

### Complications

Procedure-related complications occurred in 193 patients (45.4%). The most common complication was cement leakage; no patient developed a permanent neurological deficit. Complication rates did not differ significantly among the zone groups (*p* = 0.195) (Table 2).

### Linear regression

In the multivariable linear regression analysis (Table 3), dominant cement zone remained the strongest independent predictor of pain reduction (*β* = 0.545; 95% CI for B: 0.751–0.951; *p* < 0.001). Preoperative VAS score, pooled AO fracture severity (A3–A4 vs A1–A2), and cement volume were also independently associated with change in VAS. Serum calcium reached statistical significance but was interpreted cautiously given its small apparent effect size. Age, sex, fracture level, and NLR were not independently associated with pain reduction. The model showed a strong fit (R = 0.851), explaining 72.4% of the variance in change in VAS (R² = 0.724; adjusted R² = 0.718). Overall model significance was confirmed [F(9,377) = 109.848; *p* < 0.001], with a standard error of estimate of 0.733. The Durbin–Watson statistic was 1.666.

### Multivariable logistic regression (outcome: change in VAS > 4)

In the multivariable logistic regression analysis (Table 4), dominant cement zone remained a strong independent predictor of clinically meaningful pain improvement (overall zone effect: Wald = 75.033, *p* < 0.001). Compared with Zone 4, Zones 1, 2, and 3 were associated with significantly lower odds of achieving clinically meaningful pain reduction. Preoperative VAS score and cement volume were also independently associated with clinically meaningful improvement. Pooled AO fracture severity was independently associated with the outcome, whereas sex, age, fracture level, NLR, and serum calcium were not. Overall model fit was good, with a −2 log likelihood of 195.741, Cox & Snell R² of 0.515, and Nagelkerke R² of 0.728. The Hosmer–Lemeshow test was non-significant (*χ*² = 4.872, df = 8, *p* = 0.771), indicating adequate calibration. The model correctly classified 88.1% of cases overall.

**Table 4:** Multivariable logistic regression for clinically meaningful pain improvement (change in VAS > 4).

Variable	Comparison	B	S.E.	Wald	*p*-value	OR (ExpB)	95% CI (lower–upper)
Sex	Female vs. male	−0.365	0.430	0.719	0.397	0.695	0.299–1.613
Age	Per 1-year increase	−0.006	0.018	0.119	0.731	0.994	0.958–1.030
Fracture level	Lumbar vs. thoracic	0.780	0.409	3.628	0.057	2.181	0.978–4.866
AO severity (pooled)	A3–A4 vs. A1–A2	−1.528	0.424	13.004	<0.001	0.217	0.095–0.498
Cement zone (overall)	df = 3			75.033	**<0**.**001**		
Zone 1	Zone 1 vs Zone 4 (ref)	−6.265	1.288	23.667	**<0**.**001**	0.002	0.000–0.024
Zone 2	Zone 2 vs Zone 4 (ref)	−5.533	0.843	43.054	**<0**.**001**	0.004	0.001–0.021
Zone 3	Zone 3 vs Zone 4 (ref)	−2.214	0.771	8.239	**0**.**004**	0.109	0.024–0.495
Preoperative VAS	Per 1-unit increase	1.102	0.276	15.903	**<0**.**001**	3.010	1.751–5.173
NLR	Per 1-unit increase	−0.029	0.034	0.737	0.391	0.971	0.908–1.039
Cement volume	Per 1-unit increase	0.595	0.290	4.199	**0**.**040**	1.813	1.026–3.202
Serum calcium	Per 1-unit increase	0.011	0.010	1.221	0.269	1.011	0.992–1.031

Logistic regression was used. AO fracture morphology was analyzed using pooled severity categories (A1–A2 = less severe; A3–A4 = more severe) to avoid sparse categories and was included in the model. VAS, visual analog scale; NLR, neutrophil-to-lymphocyte ratio.

The bold values indicate statistically significant results, defined as *p* < 0.05.

## Discussion

In this large single center cohort of patients with osteoporotic vertebral compression fractures treated with unipedicular percutaneous vertebroplasty, we found that intravertebral cement distribution, categorized by a simple four zone mediolateral scheme, was strongly associated with pain improvement. Both the unadjusted comparisons and the multivariable models consistently showed that the dominant cement zone remained an independent predictor of the magnitude of pain reduction and of achieving a clinically meaningful response. These findings support the concept that vertebroplasty outcomes are influenced not only by whether cement is injected, but also by where cement predominantly accumulates within the vertebral body (20, 21).

Since the initial description of vertebroplasty, substantial debate has persisted regarding its clinical efficacy across different patient subgroups and fracture chronicity. Early sham controlled trials reported no significant advantage of vertebroplasty over placebo for pain and function at short term and mid term follow up in mixed populations that included subacute and chronic fractures (20, 21). In contrast, other randomized trials and prospective studies have suggested that carefully selected patients, particularly those with acute severe pain and imaging confirmation of an unhealed fracture, may experience clinically important benefit (17, 18). In our cohort, we observed a marked reduction in VAS after the procedure, which aligns with the broader literature indicating that vertebral augmentation can provide rapid analgesia in many real world settings, while the magnitude of benefit may depend on patient selection and fracture characteristics (22, 23).

The biological rationale for an association between cement location and pain relief is plausible. Pain in osteoporotic compression fractures is thought to be driven by micro motion at the fracture site, instability of trabecular microarchitecture, and inflammatory and nociceptive input from damaged bone and adjacent structures. Cement injected into the vertebral body can reduce painful micromotion by stabilizing the fractured trabeculae and redistributing loads. Biomechanical work has shown that both cement volume and the pattern of cement spread affect stiffness restoration, load transfer, and stress concentrations within the treated vertebra and at adjacent levels (24–26).

Several clinical and imaging based studies have similarly suggested that more central, homogeneous, or strategically distributed cement is associated with better mechanical restoration and sometimes with improved clinical outcomes, whereas eccentric or limited spread may result in less effective stabilization (27, 28). Our zonal approach adds a pragmatic framework to this concept by reducing a complex three dimensional phenomenon into a reproducible categorical descriptor that can be applied in routine postoperative imaging review.

Unipedicular vertebroplasty is increasingly used because it can shorten procedural time and reduce radiation exposure, while achieving clinical results comparable to bipedicular approaches in many studies (29–31). A key technical concern with unipedicular access is whether cement can sufficiently spread across the vertebral body to provide effective stabilization, particularly toward the contralateral side. Earlier procedural series emphasized the importance of achieving adequate mediolateral spread, sometimes using the criterion of reaching the vertebral midline or central region as a marker of technical success (32). In this context, our finding that dominant zonal location is strongly linked with pain response reinforces the idea that the quality of cement distribution, not only the choice of uni vs. bipedicular access, may be a major determinant of clinical benefit.

Cement volume correlated positively with pain improvement in our dataset and also remained significant in both the linear and logistic models, although with smaller effect sizes than the dominant zone variable. This is consistent with literature indicating that cement volume can influence vertebral stiffness and stability, but that increasing volume may yield diminishing returns and can increase leakage risk (24, 28). Importantly, cement volume and cement distribution are not independent in practice: larger volumes may facilitate broader spread, and operators may inject more cement when distribution appears insufficient. Therefore, the independent association of dominant zone after adjustment suggests that distribution captures a clinically relevant aspect of the procedure that is not fully explained by volume alone, although residual confounding remains possible in retrospective analyses.

AO fracture type was an independent predictor in our linear regression, while the logistic model indicated instability around the fracture type contrasts, likely driven by the very small number of A4 fractures and quasi separation. In general, morphology influences biomechanics and may affect both technical feasibility of cement spread and clinical response (23, 33). Baseline pain severity, reflected by preoperative VAS, was a robust predictor of achieving a clinically meaningful improvement. This is commonly observed in pain intervention studies because higher baseline scores allow greater absolute change, but it may also reflect that patients with severe acute pain and active fracture edema represent a subgroup more likely to benefit from augmentation (20–22).

We observed procedure related complications in 45.4% of patients, with cement leakage being the most frequent event and no permanent neurological deficit. The relatively high complication rate should be interpreted in the context of radiographic detection methods, since many cement leakages were imaging-detected and clinically silent. Studies using sensitive imaging, particularly computed tomography, often report high radiographic leakage rates, most of which are clinically silent (27, 28). Systematic reviews and meta analyses generally show that leakage is common, while severe neurological or pulmonary embolic complications are rare (28, 29). The absence of between zone differences in complication rates in our analysis suggests that the dominant zonal location, as defined here, is more closely linked to efficacy than to safety, although a more granular leakage classification by route and severity could reveal patterns not captured by a binary complication variable.

In our cohort, laboratory markers contributed little to the main clinical message. NLR was not independently associated with pain improvement in either multivariable model, while serum calcium reached statistical significance only in the linear model with a very small effect size and was not significant in the logistic model. Accordingly, these findings should be interpreted cautiously and considered secondary to the primary association between cement distribution zone and pain outcomes.

Recent studies also support the view that cement distribution is not merely a radiographic descriptor but a clinically relevant procedural feature in unilateral vertebral augmentation. In a 2025 study of percutaneous curved kyphoplasty stratified by Genant grade, Yang et al. reported that patients with moderate-to-severe OVCF showed superior early analgesic response together with distinctive cement distribution characteristics, reinforcing the interaction between fracture morphology, cement spread, and early clinical outcome (34). Likewise, a 2025 randomized study by Fuguo et al. comparing two unilateral kyphoplasty techniques showed that unilateral approaches may differ in cement distribution behavior and that these technical differences can translate into differences in short- and midterm outcomes (35). Taken together with our findings, these data support the concept that, in unipedicular vertebral augmentation, the spatial pattern of cement distribution deserves attention as a practical procedural quality marker rather than being viewed solely as a postoperative imaging finding.

Taken together, our results support a clinically intuitive message: in unipedicular vertebroplasty, achieving an advantageous pattern of intravertebral cement spread may matter as much as, or more than, the absolute amount of cement injected. This aligns with the broader vertebral augmentation literature in which technical success is increasingly framed by distribution quality rather than simply by unipedicular or bipedicular access (29, 30, 32). Because our zonal scheme is simple and based on routine imaging, it could be incorporated into procedural reporting and quality improvement efforts, and it may help standardize future comparative research on cement distribution.

From a practical perspective, our findings suggest that achieving a more favorable mediolateral cement distribution—particularly toward the outer zones—may be considered a procedural target during unipedicular vertebroplasty. Several technical factors may influence cement spread within the vertebral body. First, the trajectory of the working cannula is critical; positioning the needle tip closer to the midline or slightly beyond it may facilitate more balanced or contralateral cement distribution. Second, cement viscosity at the time of injection plays a key role, as lower-viscosity cement may allow wider trabecular penetration, whereas higher-viscosity cement may result in more localized accumulation. Third, controlled and gradual injection under continuous fluoroscopic monitoring may help guide cement flow and reduce the risk of premature accumulation in a limited zone.

In addition, subtle adjustments in injection pressure and timing may influence the final distribution pattern. Rather than a fixed protocol, these factors should be dynamically adapted intraoperatively according to real-time imaging feedback. Although our study did not directly compare thoracic and lumbar vertebrae in terms of optimal injection strategy, the proportional nature of the four-zone classification allows it to be applied across different vertebral levels. Therefore, the classification may serve as a simple intraoperative reference to assess whether cement distribution is likely to achieve effective biomechanical stabilization and pain relief.

Although our findings demonstrate a strong association between cement distribution and early postoperative pain relief, the long-term prognostic implications of the four-zone classification remain uncertain. Cement distribution patterns may potentially influence longer-term outcomes such as vertebral refracture, adjacent vertebral fractures, and functional recovery by affecting load distribution and biomechanical stability within the treated vertebra. Previous studies have suggested that more homogeneous and strategically distributed cement may reduce stress concentration and possibly lower refracture risk. However, these hypotheses could not be directly evaluated in the present study due to the lack of standardized long-term follow-up data. Future prospective studies with longitudinal follow-up are warranted to determine whether zonal cement distribution has sustained predictive value beyond early pain outcomes.

This study has several limitations. First, its retrospective single-center design introduces potential selection and measurement bias. Several clinically important confounders could not be fully accounted for, including fracture chronicity/acuity, MRI bone marrow edema status, osteoporosis severity (e.g., T-scores), anti-osteoporotic therapy, preoperative analgesic use, and variability in postoperative follow-up timing and VAS documentation. These unmeasured factors may have influenced both baseline pain and postprocedural response, resulting in residual confounding. In addition, 38 patients were excluded from the logistic regression because of missing data in one or more covariates; because the missingness mechanism could not be formally determined, complete-case analysis may have introduced selection bias. Although procedures were performed using a standardized institutional technique, detailed technical variables such as exact cement mixing characteristics, injection pressure or speed, and other operator-level nuances were not uniformly available and therefore could not be analyzed separately.

The four-zone classification was based on visual assessment of the dominant mediolateral cement location on postoperative imaging rather than formal volumetric quantification, which may have introduced observer-related bias; however, interobserver agreement was high (Cohen's kappa = 0.84). The marked imbalance in group sizes, particularly the small number of patients in Zone 1, may have reduced the precision and stability of subgroup estimates, especially in the logistic regression analysis, and therefore findings involving Zone 1 should be interpreted cautiously. Although pooled AO severity categories were used to improve model stability, the rarity of A4 fractures limited fracture-type–specific inference. In addition, although the regression models showed good apparent fit, overfitting remains possible given the number of predictors, and external validation is needed before clinical application. Finally, this study focused on short-term postoperative pain relief and did not include standardized long-term follow-up data on pain, functional outcomes, vertebral refracture, or adjacent vertebral fractures; therefore, the long-term prognostic value of the four-zone classification remains uncertain. Another important limitation is the lack of standardized long-term follow-up data, including mid- and long-term pain scores, functional outcomes, and vertebral refracture or adjacent fracture rates. Therefore, the findings of this study are limited to early postoperative outcomes, and the long-term prognostic value of the four-zone classification system could not be determined.

Prospective studies could test whether intentionally targeting cement spread to achieve specific zonal patterns improves outcomes, ideally with standardized imaging protocols, blinded distribution scoring, and stratification by fracture age and MRI edema. Integration of quantitative imaging measures, such as volumetric cement distribution or midline crossing metrics, may also help refine the mechanistic link between cement location and analgesic response.

## Conclusion

In conclusion, in this large single center cohort of patients with osteoporotic vertebral compression fractures treated with unipedicular percutaneous vertebroplasty, we observed substantial postoperative pain reduction and demonstrated that the dominant intravertebral cement zone, defined using a simple four zone mediolateral scheme, was a strong and independent predictor of pain improvement and of achieving clinically meaningful response. Cement volume and baseline pain severity also contributed to outcomes, whereas NLR and fracture level were not independently associated with pain reduction. Although cement leakage was common, no permanent neurological deficit occurred and complication rates did not differ across zones. These findings suggest that optimizing cement distribution within the vertebral body may represent a practical and potentially modifiable technical target to improve clinical results in unipedicular vertebroplasty, warranting prospective validation with standardized imaging and follow up.

## Data Availability

The raw data supporting the conclusions of this article will be made available by the authors, without undue reservation.
