# Supplementary material for: Effect of dominant cement distribution zone on pain relief after unipedicular percutaneous vertebroplasty
Source: Front Surg. 2026 Apr 8;13:1806822. doi: 10.3389/fsurg.2026.1806822 (PMC13099904; doi:10.3389/fsurg.2026.1806822)
Supplement: Supplementary file 1 [file Table1.docx]

**Correlation Table.** Correlations between change in VAS and NLR, cement volume, and serum calcium.

| **Variable** | **Correlation with change in VAS (r)** | **p-value** |
| --- | --- | --- |
| NLR | -0.061 | 0.218 |
| Cement volume | 0.408 | **<0.001** |
| Serum calcium | 0.088 | 0.079 |

Spearman correlation test was used. Bonferroni correction was applied for the three correlation analyses (adjusted significance threshold p<0.0167, i.e., 0.05/3). VAS: Visual Analog Scale, NLR: **Neutrophil-to-lymphocyte ratio**
